# Conditionally Replicating Adenovirus Expressing TIMP2 Increases Survival in a Mouse Model of Disseminated Ovarian Cancer

**DOI:** 10.1371/journal.pone.0025131

**Published:** 2011-10-12

**Authors:** Sherry W. Yang, Diptiman Chanda, James J. Cody, Angel A. Rivera, Reinhard Waehler, Gene P. Siegal, Joanne T. Douglas, Selvarangan Ponnazhagan

**Affiliations:** 1 Department of Pathology, The University of Alabama at Birmingham, Birmingham, Alabama, United States of America; 2 Department of Medicine, The University of Alabama at Birmingham, Birmingham, Alabama, United States of America; 3 Department of Obstetrics & Gynecology, The University of Alabama at Birmingham, Birmingham, Alabama, United States of America; Baylor College of Medicine, United States of America

## Abstract

Ovarian cancer remains difficult to treat mainly due to presentation of the disease at an advanced stage. Conditionally-replicating adenoviruses (CRAds) are promising anti-cancer agents that selectively kill the tumor cells. The present study evaluated the efficacy of a novel CRAd (Ad5/3-CXCR4-TIMP2) containing the CXCR4 promoter for selective viral replication in cancer cells together with TIMP2 as a therapeutic transgene, targeting the matrix metalloproteases (MMPs) in a murine orthotopic model of disseminated ovarian cancer. An orthotopic model of ovarian cancer was established in athymic nude mice by intraperitonal injection of the human ovarian cancer cell line, SKOV3-Luc, expressing luciferase. Upon confirmation of peritoneal dissemination of the cells by non-invasive imaging, mice were randomly divided into four treatment groups: PBS, Ad-ΔE1-TIMP2, Ad5/3-CXCR4, and Ad5/3-CXCR4-TIMP2. All mice were imaged weekly to monitor tumor growth and were sacrificed upon reaching any of the predefined endpoints, including high tumor burden and significant weight loss along with clinical evidence of pain and distress. Survival analysis was performed using the Log-rank test. The median survival for the PBS cohort was 33 days; for Ad-ΔE1-TIMP2, 39 days; for Ad5/3-CXCR4, 52.5 days; and for Ad5/3-CXCR4-TIMP2, 63 days. The TIMP2-armed CRAd delayed tumor growth and significantly increased survival when compared to the unarmed CRAd. This therapeutic effect was confirmed to be mediated through inhibition of MMP9. Results of the *in vivo* study support the translational potential of Ad5/3-CXCR4-TIMP2 for treatment of human patients with advanced ovarian cancer.

## Introduction

Ovarian cancer is the leading cause of gynecological cancer death in the U.S. [1]. The significant morbidity and mortality in patients with this group of tumors is attributed to the fact that patients present only with nonspecific symptoms at the early stages, which precludes its diagnosis [2]. The majority of the patients are diagnosed at Stage III or beyond, where the cancer has disseminated throughout the peritoneal cavity, which leads to a poor prognosis. Current therapies have limitations, as the five-year survival rate has remained unchanged at 50% over the past four decades [3]. Thus, novel and effectively targeted therapies for disseminated disease are urgently needed.

Conditionally replicating adenoviruses (CRAds) are a promising new class of therapeutics, as these viruses have the potential to selectively and self-perpetually replicate and lyse cancer cells to eradicate the entire tumor [4]. Clinical trials with CRAds have so far demonstrated the safety of oncolytic adenoviruses. However, modest therapeutic efficacy with CRAds suggests further optimization for effective clinical translation is required [5]. One strategy of augmenting antitumor efficacy utilizes the CRAd as a platform for the delivery of a therapeutic transgene, in addition to their oncolytic potential [6]. To that end, we hypothesized that efficacy of a replicating adenovirus could be improved for the treatment of ovarian cancer by arming with a gene that acts on the host microenvironment, as the interplay between the cancer cells and its microenvironment has been known to modulate tumor progression [7], [8], [9].

Matrix metalloproteinases (MMPs) are a diverse family of proteases capable of degrading multiple components of the extracellular matrix (ECM) [10]. They are one of the ideal targets for ovarian cancer therapy, as their dysregulation has been shown to contribute to the promotion of tumor growth, angiogenesis, invasion and metastasis [11]. In particular, MMP-2 and MMP-9 are consistently upregulated in ovarian cancer and are associated with poor prognosis [12], [13]. Important regulator of MMPs are their endogenous inhibitors, the tissue inhibitor of metalloproteinases (TIMP), a family of small secreted proteins that act by binding directly to active MMPs thereby inhibiting their action. To date, four mammalian TIMPs have been identified [14]. Among the TIMP family members, TIMP-2 is unique in its ability to inhibit tumor growth and angiogenesis through pathways also independent of MMP inhibition [15], [16], [17]. We hypothesized that the production of TIMP2 from virus infected cells should bind and inhibit excess extracellular MMPs and thereby inhibit tumor progression through both MMP-dependent and MMP-independent pathways.

Previously, we developed and determined the therapeutic potential of a TIMP2-armed CRAd, Ad5/3-CXCR4-TIMP2, for ovarian cancer therapy [18]. Transductional selectivity of the CRAd was achieved by genetically replacing the Ad5 knob with the Ad3 knob, which circumvents the coxsackie and adenovirus receptor (CAR) and redirects binding to the Ad3 receptor, which is more abundantly expressed on ovarian cancer cells [19], [20]. The CXCR4 promoter was used to mediate tumor selective replication of the vector, as it exhibits superior activity in both established human ovarian cancer cells and patient-derived primary tumor tissues [18]. Further, activity of CXCR4 promoter is minimal in the liver, the major organ known to modulate systemically delivered adenoviral vectors [21]. The TIMP2 gene was incorporated to inhibit tumor progression. We recently validated *in vitro* that Ad5/3-CXCR4-TIMP2 produced functional TIMP2, as indicated by the inhibition of the enzymatic degradation of gelatin by active MMPs [18]. For the TIMP2-armed CRAd to be efficacious, it was important that the expression of TIMP2 did not impair viral replication or its oncolytic potency. We have recently demonstrated that both viral replication and oncolytic potency were not compromised in both SKOV3.ip1 and OV-4 human ovarian cancer cells by arming with TIMP2 [18]. Further, we demonstrated that there was selectivity in replication with the CXCR4 promoter, as indicated by a higher level of replication in the ovarian cancer cells, when compared to control fibroblasts. Towards translating this virus into the clinic, we further employed the usage of an *ex vivo* model system to further examine viral replication in tumors tissues obtained from patients with confirmed stage III and IV ovarian cancer. Results of this study indicated a consistently high-level replication with Ad5/3-CXCR4-TIMP2 [18]. Collectively, these data were very encouraging, suggesting that Ad5/3-CXCR4-TIMP2 might be effective for the treatment of advanced ovarian cancer. In the present study, we sought to determine the therapeutic efficacy of Ad5/3-CXCR4-TIMP2 in a murine orthotopic model of disseminated ovarian cancer. Tumor progression was monitored through non-invasive *in vivo* imaging. Results of the present study demonstrated a significant increase in survival following treatment with Ad5/3-CXCR4-TIMP2, suggesting its potential for clinical translation.

## Materials and Methods

### Cells

The firefly luciferase–expressing ovarian adenocarcinoma cell line SKOV3-luc was kindly provided by Dr. R. Negrin (Stanford Medical School, Stanford, CA). The A549 human lung carcinoma cell line was purchased from the American Type Culture Collection (ATCC; Manassas, VA). Both cell lines were cultured in a 1∶1 mixture of Dulbecco's modified Eagle medium (DMEM) and Ham's F-12 medium, supplemented with 10% (v/v) heat-inactivated fetal bovine serum (FBS; Invitrogen, Carlsbad, CA), L-glutamine (2 mM), penicillin (100 U/ml) and streptomycin (100 µg/ml), at 37°C in a humidified atmosphere and at 5% CO_2_. Media and supplements were purchased from Mediatech (Herndon, VA).

### Construction and production of the viruses

Ad-ΔE1-TIMP2 is an E1-, E3-deleted replication-deficient Ad5 vector which expresses TIMP2 under the control of the CMV promoter [22]. Ad5/3-CXCR4, is a CRAd with the CXCR4 promoter inserted into the E1A position along with a tropism-modified fiber, containing the Ad5 fiber shaft and tail that has had the knob domain replaced with that of the Ad3 virus [23]. The Ad5/3-CXCR4-TIMP2 was constructed using a full-length human TIMP2 cDNA as described earlier [18]. The replication-deficient adenovirus, Ad-ΔE1-TIMP2 was propagated in 293 cells. The CRAds, Ad5/3-CXCR4 and Ad5/3-CXCR4-TIMP2 were amplified in the A549 cell line. All viruses were purified by two rounds of cesium chloride density centrifugation. The titers of viral particles and infectious units were determined as previously described [24].

### Orthotopic ovarian cancer model and vector treatment

Female BALB/c nu/nu mice of 6 to 8 weeks of age were obtained from the National Cancer Institute-Frederick Animal Production Area (Frederick, MD) and quarantined for 2 weeks. Mice were kept under pathogen-free conditions according to the American Association for Accreditation of Laboratory Animal Care guidelines. Animal protocols were reviewed and approved by the Institutional Animal Care and Use Committee of the University of Alabama at Birmingham. *In vivo* optical imaging was performed at the small animal imaging core facility in the Comprehensive Cancer Center of the University of Alabama at Birmingham. On day 0, mice were injected intraperitoneally (i.p.) with 5×10^6^ SKOV3-luc cells in 250 µl PBS and imaged for bioluminescence to obtain baseline readings. Eight days later, mice were reimaged to ensure the implantation of the tumors and were then randomly divided into four groups: PBS, Ad-ΔE1-TIMP2, Ad5/3-CXCR4, and Ad5/3-CXCR4-TIMP2, with n = 10 per group. Each mouse was injected i.p. with 2×10^8^ IU of the given virus in 200 µl PBS, or PBS alone. All mice were imaged for bioluminescence weekly after tumor cell injection to monitor tumor growth. All mice were followed daily to record survival. Survival time reflects the time required for the animals to reach both of the following parameters. First, the clinical evidence of pain or distress. Second, any of the pre-determined measureable endpoints including: weight loss exceeding 15%, or weight gain exceeding 20% of the body weight. The animal weight was measured at day 0 and remeasured every 3 days until the end of the experiment. Survival data were plotted on a Kaplan-Meier curve, and different groups were compared using the Log-rank (Mantel-Cox) test (GraphPad Prism Software v.5).

### Bioluminescence imaging to detect *in vivo* luciferase expression

Mice were imaged on day 0 for bioluminescent signal after injection of SKOV3-luc cells to obtain a baseline reading of tumor burden. Imaging was performed again before initial treatment on day 8 and then weekly until mice were sacrificed. Briefly, 150 mg/kg D-luciferin was injected i.p., and mice were anesthetized with isoflurane gas anesthesia and placed in a light-tight chamber. The photographic (gray-scale) reference image was obtained at 10 min after D-luciferin injection, and the bioluminescent image was collected immediately thereafter. Images were obtained with a CCD cooled to −120°C, using the IVIS −100 Imaging System (Xenogen Corp., Alameda, CA), with the field of view set at a 25 cm height. The bioluminescent images used exposures ranging from 1 to 10 s, 1 f/stop, 4 binning, and open filter. Data acquisition software was calibrated so that no pixels were saturated during image collection. The bioluminescent and gray-scale images were overlaid using LivingImage software. Living Image Software (Xenogen, Alameda, CA) was also used to obtain a pseudocolor image representing bioluminescence intensity (blue, least intense, and red, most intense). Regions of interest were drawn around the i.p. tumors, and the total counts (photons) were summed for the entire tumor area. The total counts were normalized to image acquisition time (photons/sec). Statistical differences of tumor size among groups were assessed by the Student's t test. A *p* value of <0.05 was considered statistically significant. Data were presented as mean values ±SD.

### Immunohistochemistry

Mice were sacrificed at high tumor burden by predetermined parameters defined as above. At time of sacrifice, necropsies were performed on 3 mice from each treatment group, from which tumors and major organs were harvested and fixed into paraffin blocks. Briefly, paraffin sections of xenografted human ovarian tumors were deparaffinized in xylene and hydrated through graded alcohol. Antigen retrieval was performed in citrate buffer (pH 6.0), under steam for 20 min. Sections were cooled to room temperature, and endogenous peroxidase was removed using 0.3% H_2_O_2_ in methanol for 30 min and blocked with 5% BSA for 30 min. Tissue sections were then incubated with primary antibodies overnight at 4°C. Sections were washed in Phosphate-Buffered Saline containing 0.05% Tween-20 (PBST) and again incubated at room temperature with biotin-conjugated goat anti-goat/anti-mouse secondary antibody for 2 h. After washing, sections were incubated with streptavidin-conjugated horseradish peroxidase for 1 h at room temperature. After another wash with PBST, immunodetection was performed using 3,3′-diaminobenzidine-H_2_O_2_ (Vector Labs) and counterstained with hematoxylin.

## Results

### Arming with TIMP2 delayed tumor growth *in vivo*

**Figure 1:**
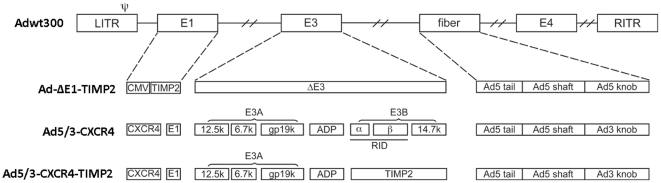
Genomic organization of armed CRAd and control viruses. Adwt300, is the wild- type Ad5 virus. Ad-ΔE1-TIMP2 is an E1-, E3-deleted replication-deficient Ad5 vector which expresses TIMP2 under the control of the CMV promoter in E1. Ad5/3-CXCR4 is an unarmed CRAd with a CXCR4 promoter in E1 to limit viral replication to the cancer cells in conjunction with a Ad5/3 modified fiber that contains the shaft and tail of Ad5 fiber and the Ad3 fiber knob. Ad5/3-CXCR4-TIMP2 is a CRAd with the CXCR4 promoter in E1, armed with TIMP2 in the E3B region and the aforementioned F5/3 modified fiber.

**Figure 2:**
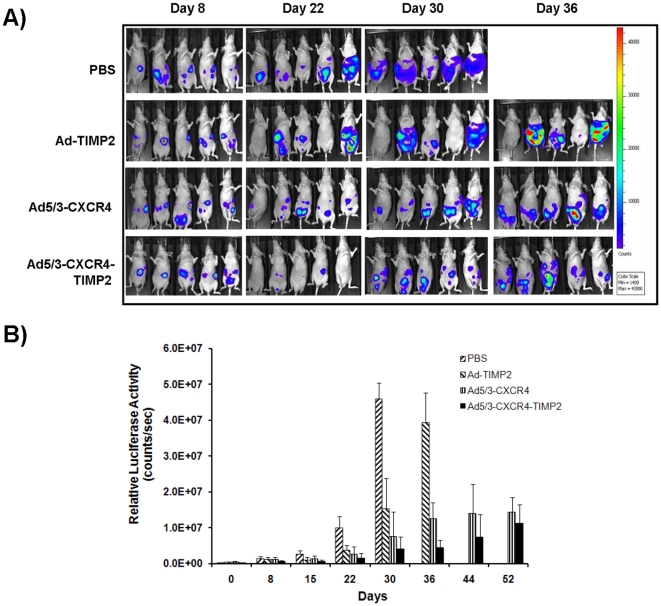
Bioluminescent imaging of mice before and after treatment. Disseminated tumors were established by injecting 5×10^6^ SKOV3-luc cells i.p. into female athymic nude mice on day 0. On day 8, mice were treated with either an i.p. injection of PBS, or 2×10^8^ IU of Ad-ΔE1-TIMP2, Ad5/3-CXCR4, and Ad5/3-CXCR4-TIMP2, respectively. Bioluminescence levels were measured weekly. (A), pseudocolor image representing bioluminescence intensity on days 8, 22, 30 and 36. (B), bioluminescence intensity from the abdominal region was quantitated and normalized, and the mean photon counts per second for each group are shown for the given days. Data reported as the mean ± SD for each treatment group. *p<0.05.

To evaluate the therapeutic efficacy of Ad5/3-CXCR4-TIMP2 in a clinically relevant *in vivo* model, we generated orthotopically disseminated ovarian cancer by injecting female nude mice with human SKOV3-luc cells intraperitoneally (i.p.). A luciferase-expressing stable cell line was used to monitor the therapeutic efficacy of treatments by noninvasive imaging *in vivo*. Eight days following tumor cell administration, mice were re-imaged to ensure implantation of the tumors and divided into four treatment groups, with 10 mice per group. The control groups received PBS, a replication deficient vector expressing TIMP2 (Ad-ΔE1-TIMP2), or a CXCR4 promoter-regulated CRAd with a chimeric Ad5/3 fiber (Ad5/3-CXCR4). The therapeutic group received a TIMP2-armed CXCR4 promoter based CRAd with a chimeric Ad5/3 fiber (Ad5/3-CXCR4-TIMP2) (Fig. 1). All mice were imaged after i.p. injections of D-luciferin on a weekly basis. A panel of representative images is shown in Fig. 2A, while quantitative determination of bioluminescent signals is shown in Fig. 2B. Low bioluminescent signals of SKOV3-luc cells were detected as early as 8 days following tumor cell injection, and were relatively consistent between the four groups. By day 22, there was significant tumor growth in the PBS group and Ad-ΔE1-TIMP2 treated group. In contrast, groups treated with the CRAds had significant inhibition in tumor growth. The group treated with the target virus, Ad5/3-CXCR4-TIMP2, exhibited tumor regression at day 22. By day 30, there was considerable tumor burden in the PBS group, as mice exhibited a considerable increase in abdominal girth and were beginning to show signs of disease burden. In contrast, tumor growth was significantly inhibited in all groups treated with viruses (p<0.001). By day 36, the majority of the mice in the PBS group had died from a heavy tumor burden. At this time point, mice treated with Ad-ΔE1-TIMP2, began to display considerable tumor burden. However, both groups treated with CRAds had significantly less bioluminescent signal (p<0.001), indicating a slower rate of tumor development. Furthermore, tumor burden in the cohort of mice treated with Ad5/3-CXCR4-TIMP2, the armed CRAd, was significantly less when compared to the tumor burden in mice treated with both Ad5/3-CXCR4 and the unarmed CRAd (p<0.01; Fig. 2B). A similar trend in survival following treatment with different vectors was observed in two pilot studies with less animal numbers per group (data not shown). Collectively, these data suggest that both viral replication and arming with TIMP2 contributed to delaying the onset of tumor growth.

### Arming with TIMP2 increased survival

**Figure 3:**
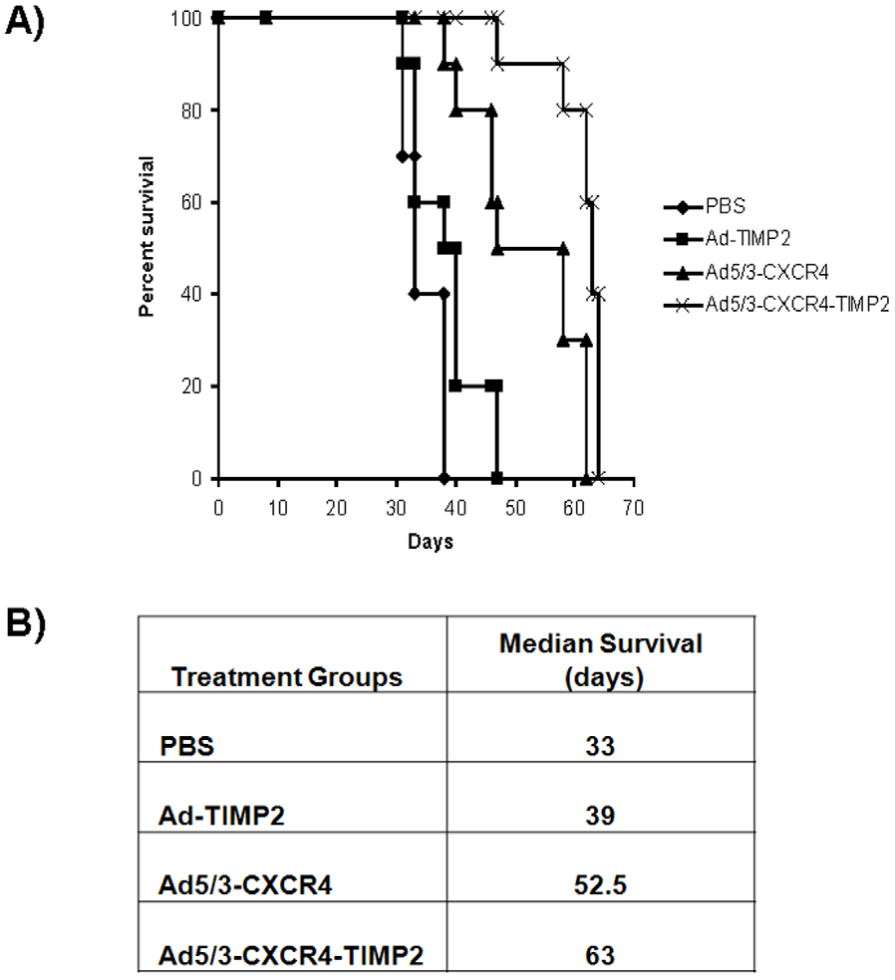
Survival analysis. Mice were monitored for survival. Survival data are plotted on a Kaplan-Meier curve (A). Treatment with Ad5/3-CXCR4-TIMP2 significantly enhanced survival when compared to treatment with Ad-ΔE1-TIMP2, Ad5/3-CXCR4, or PBS (p<0.05). (B), median survival in days for each treatment group.

All mice were then followed for survival as shown in Fig. 3A. The median survival time increased from 33 days, for the PBS cohort, to 39 days in the cohort injected with the nonreplicating vector expressing TIMP2 (Ad-ΔE1-TIMP2), this difference was statistically significant (p<0.03). The cohort treated with the unarmed CRAd (Ad5/3-CXCR4) had median survival time of 52.5 days, whereas the cohort treated with the TIMP2-armed CRAd (Ad5/3-CXCR4-TIMP2) had a median survival time of 63 days (Fig. 3B), this difference with also statistically significant (p<0.002). In summary, arming with TIMP2 appears to be useful for the treatment of disseminated ovarian cancer, as it increased survival *in vivo*.

### TIMP2 is expressed in the tumors of armed viruses

**Figure 4:**
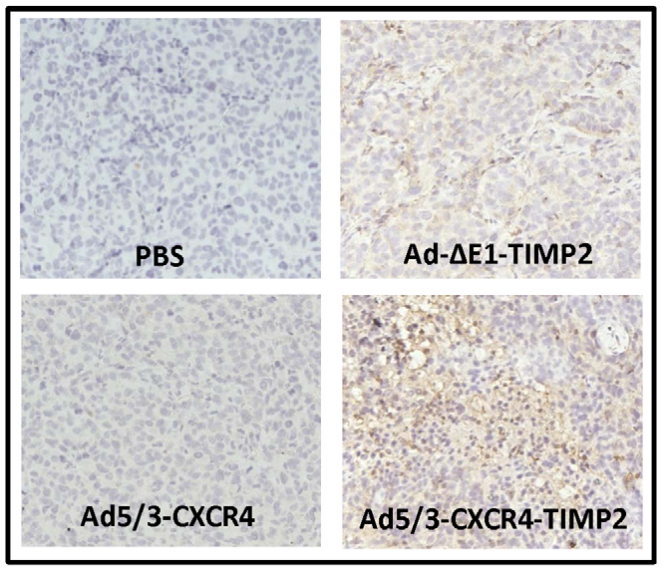
TIMP-2 expression in excised epithelial ovarian tumor. At the time of sacrifice of mice due to tumor burden, tumors were collected from indicated treatment groups and examined for expression of TIMP2 by immunohistochemistry.

To determine if the increased survival seen in the TIMP2-armed CRAd when compared to the unarmed CRAd was due to modulation of the microenvironment by TIMP2, expression of TIMP2 was first examined in tumors excised from each treatment group at autopsy. As seen in Fig. 4, tumors excised from groups treated with PBS and Ad5/3-CXCR4 were negative for TIMP2 expression. Result of the immunohistochemistry indicated that in tumors excised from cohort treated with the Ad-ΔE1-TIMP2 , TIMP2 expression was minimal and appeared limited to the periphery of the tumor. This was not unexpected, as Ad-ΔE1-TIMP2 is a non-replicating vector and was not expected to disseminate within the tumor effectively. In contrast, tumors from mice treated with Ad5/3-CXCR4-TIMP2 exhibited enhanced TIMP2 staining throughout the tumor, indicating that this oncolytic virus was effective in infecting and replicating throughout the entire tumor.

### TIMP2-armed CRAd decreased the level of active MMPs

**Figure 5:**
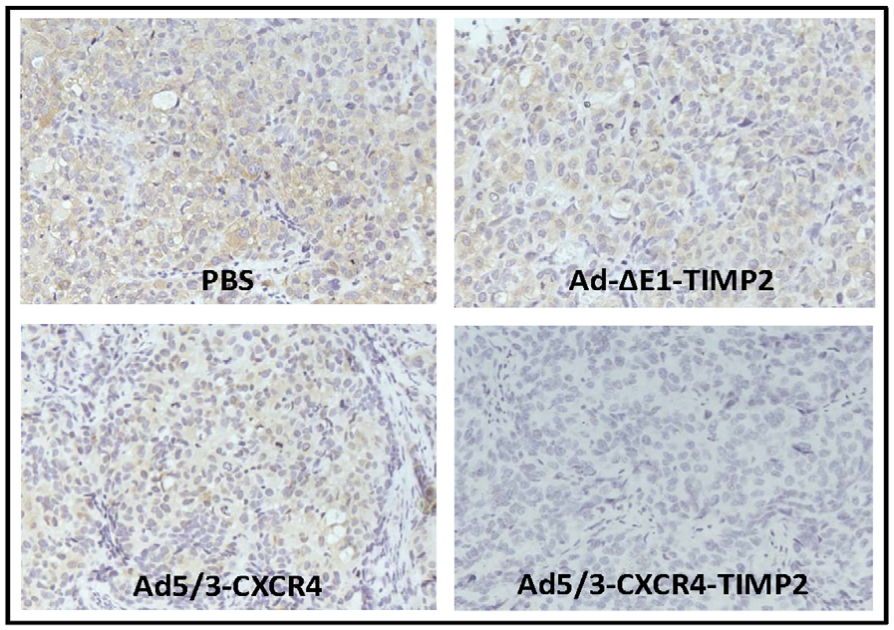
MMP-9 expression in excised epithelial ovarian tumor. At the time of sacrifice of mice due to tumor burden, tumors were collected from indicated treatment groups and examined for expression of MMP-9 by immunohistochemistry.

TIMP2 is a known inhibitor of MMP2 and MMP9. Hence, staining for active MMP2 and MMP9 was performed to determine if the levels were affected. Results of this analysis indicated that tumors from groups treated with PBS, Ad5/3-CXCR4, and Ad-ΔE1-TIMP2, all exhibited high levels of active MMP2. In contrast, tumors from the group treated with Ad5/3-CXCR4-TIMP2 had very low levels of MMP2. An identical pattern was also detected in MMP9 expression by immunohistochemistry. Groups treated with PBS, Ad5/3-CXCR4, and Ad-ΔE1-TIMP2, all exhibited immunoreactivity for active MMP9. In contrast, tumors from the group treated with Ad5/3-CXCR4-TIMP2 had very low levels of active MMP9 expression (Fig. 5). These data suggest that TIMP2 secreted by the armed CRAd was also effective in inhibiting MMP2 and MMP9 activities.

### TIMP2-armed CRAd inhibited angiogenesis

**Figure 6:**
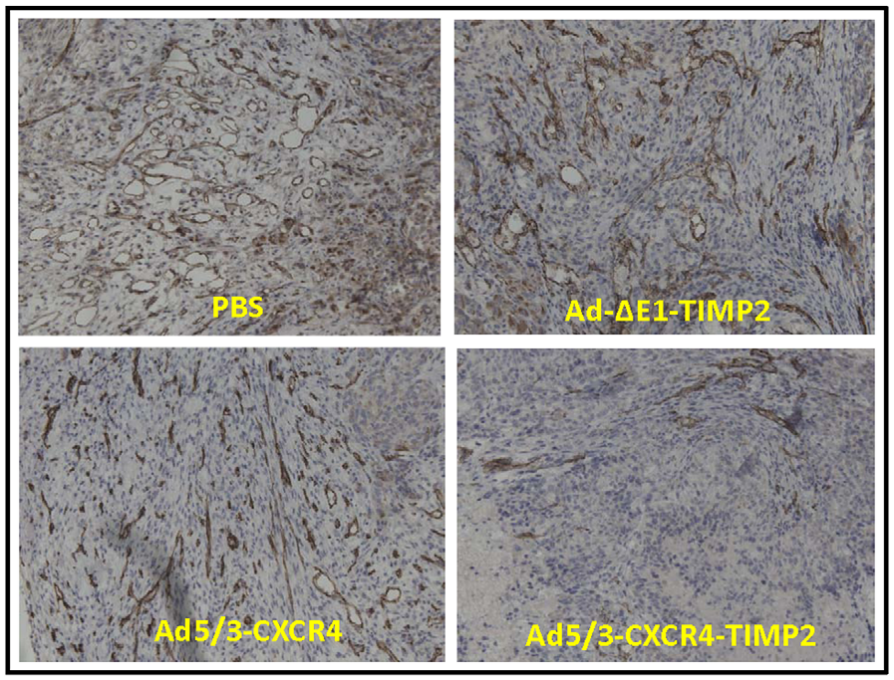
CD31 expression in excised epithelial ovarian tumor. Tumor conglomerates in the abdominal area were collected during sacrifice of the animals from indicated treatment groups and fixed. Five µM sections of paraffin blocks of tumor tissues were stained with human CD31 antibody to detect blood vasculature.

Finally, tumors were examined for expression of CD31, a marker for angiogenesis. Cohorts treated with PBS, Ad-ΔE1-TIMP2 , and Ad5/3-CXCR4, had large diameter vessels with minimal immunoreactivity lining the endothelial walls when compared to the cohorts treated with Ad5/3-CXCR4-TIMP2 (Fig. 6).

## Discussion

The importance of the interplay between tumor cells and the microenvironment in modulating tumor progression has long been recognized [25]. In the pathogenesis of ovarian cancer, MMP-2 and MMP-9 are consistently upregulated [12]. Furthermore, increased levels of these MMPs correlated negatively with survival as they are associated with advanced stage, high-grade ascites and positive (metastatic) lymph node [13]. These data suggest that MMPs are valid therapeutic targets for the treatment of ovarian cancer. A key regulator of MMPs is their endogenous inhibitors, TIMPs. Four mammalian TIMPs have been identified thus far, among them TIMP2 has been most extensively studied due to its ability to inhibit tumor growth and angiogenesis by a variety of mechanisms independent of direct MMP-inhibition [15], [16], [17]. TIMP2 can inhibit endothelial cell proliferation through induction of protein tyrosine phosphatase activity [17]. TIMP2 also inhibits tumor growth and angiogenesis by increasing activity of mitogen-activated protein kinase phosphatase 1 [15]. In an effort to develop a successful CRAd geared toward the treatment of disseminated ovarian cancer, we proposed that arming it with TIMP2 would augment the therapeutic efficacy of the CRAd by inhibiting tumor progression through both MMP-dependent and MMP-independent pathways.

In our recent study, we characterized and evaluated the efficacy of Ad5/3-CXCR4-TIMP2 *in vitro*. We confirmed that TIMP2 expression did not impair viral replication or oncolytic potency of the virus. Furthermore, selective replication was achieved with the CXCR4 promoter. Validation of Ad5/3-CXCR4-TIMP2 on primary tumor samples from five patients with confirmed stage III or IV ovarian cancer, revealed a consistent high level of replication when compared to the other control viruses. Collectively, these data suggested the potential of Ad5/3-CXCR4-TIMP2 for the treatment of advanced ovarian cancer [18].

In the present study, we sought to determine the efficacy of Ad5/3-CXCR4-TIMP2 *in vivo*. Noninvasive bioluminescent imaging provides a powerful tool that permits longitudinal monitoring of tumor growth in mice. Moreover, it allowed us to use a model system that is clinically relevant. In the majority of cases, ovarian cancer is diagnosed at an advanced stage, where the cancer has disseminated throughout the peritoneal cavity, thus, using a subcutaneous animal model to evaluate efficacy of treatment, while convenient, does not reflect the natural clinical progression of the disease. Therefore, establishing disseminated tumors with i.p. injection of SKOV3-luc cells along with imaging enabled us to use a clinically valid model system that can more accurately and effectively predict the translational value of Ad5/3-CXCR4-TIMP2. Moreover, several studies have shown sensitivity and validity of bioluminescent imaging in i.p. tumor models [26], [27], [28].

Treatment with a TIMP2-armed CRAd significantly increased survival when compared to the unarmed CRAd, however the increase was not robust. In the present study, virotherapy was given with a single injection of low dose virus on day 8 after tumor implantation. In contrast, many *in vivo* experiments examining efficacy initiated treatment either before or on the same day of tumor injection. Moreover, the viral doses used were at least one to two logs higher and were delivered via multiple administrations of the virus [29], [30], [31], [32]. The main purpose of limiting the vector dose in the present study was to establish a safe therapeutic index. Since the nude mouse models used in the preclinical evaluation of experimental therapies of ovarian cancer do not have a functional immune system, increasing the vector dose to the highest level possible to achieve a therapeutic effect may not be appropriate for extrapolation to humans as the highest vector dose results in activation of the immune system causing grave side effects. However, from data in this study, it is conceivable that with a higher dose or a multiple dosing regimen, therapeutic efficacy could be further enhanced. One potential downside with repeat administration of the virus is that immunity could become a significant problem and hamper therapeutic efficacy. Recent publication by *Hemminki et al* suggest that even in the presence of significant immunity against Ad5, one could bypass this immunity by simply using a serotype that is less prevalent in the population, such as Ad3 and thus achieving therapeutic efficacy without the need to increase the viral dose. This principle could be easily applied to repeat administrations of virus, where if immunity develops to the serotype used, then one can simply change the backbone of the virus to a less prevalent serotype [33]. In addition, augmentation of viral efficacy could also be achieved by a multi-modality approach using radiation or chemotherapy as adjuvant therapies [34], [35], [36]. With our current platform of the virus targeting through Ad5/3 it is conceivable that it could potentially infect and replicate in human immune cells, the extent of which cannot be determined in this model, as human Adenovirus do not replicate in mouse tissue and athymic nude mice lack T cells. However this potential side-effect could be mitigated by changing the tropism of the viral platform. Perreau *et al* showed decreased infection of dendritic cells with canine adenovirus serotype 2 (CAV-2) fiber knob [37]. Thus, by switching the fiber knob from Ad5/3 to Ad-CAV2, we can prevent the transduction of dendritic cells. Furthermore, this allows to retain the CXCR4 promoters as it exhibits high activity in tumor cells.

The tumor biology, the nature of peritoneal dissemination of ovarian cancer, as well as the paradoxical roles of MMPs and TIMPs in cancer progression further adds complexity to effective treatment of the disease. It is well established that increased MMP-2 and MMP-9 expression in ovarian cancer correlates negatively with prognosis and survival [12], [13], [38], [39], [40], [41]. However, the specific roles of MMPs in ovarian cancer progression are not well defined. Studies indicate that MMP-2 is predominately important in early tumorgenesis by initiating metastasis through enhancing adhesion of ovarian cancer cells to the peritoneum [42]. Thus, therapies to block it once metastasis is established are ineffective. However, other reports support the role of MMP-9 in tumor growth and angiogenesis [43]. The roles of MMPs and TIMPS in ovarian cancer are further confounded by immunohistochemical studies looking at expression of TIMP2 in ovarian cancer tissues. While some groups have reported low expression of TIMP2 in primary ovarian tumors [38], others have seen increased TIMP2 expression in ovarian cancer tissues, suggesting that TIMP2 contributes to tumor progression, in part through the activation of pro-MMP2 and inhibition of anti-tumor activities of MMPs [39], [41]. In contrast, several animal studies utilizing viral delivery of TIMP2 have shown its utility to suppress tumor growth, migration and metastasis in various cancer models [44], [45], [46], [47], [48]. Collectively these studies indicate that perhaps effects of TIMP2 on tumor promotion or suppression are dependent on timing and the specific type of tumor. Rather than nonspecific inhibition of MMPs, it is perhaps more useful to specifically inhibit different MMPs during stages of tumor progression.

Emerging data from clinical, pathological, and molecular genetic studies suggest a new model for ovarian carcinogenesis, where ovarian cancer is classified into two distinct groups, type I and II [49]. Type I tumors are clinically indolent, as they are slow growing and generally confined to the ovary at diagnosis. In contrast, type II tumors are clinically aggressive, present at an advanced stage and are believed to arise *de novo*, as precursor lesions have not been identified. The type II tumors, accounting for 70% of ovarian cancer, are characterized by mutations of TP53 in 80% of the cases. This new data suggest that p53 could potentially be a therapeutic target to arm the virus for ovarian cancer therapy. CRAds armed with p53 have been shown *in vitro* to enhance oncolysis in cervical cancer [50] and a variety of other cancer cell lines [51]. Another potential therapeutic target is CXCR4, the only chemokine receptor found to be expressed on ovarian cancer [52]. The ligand for CXCR4 is CXCL12, which can stimulate ovarian cancer cells migration *in vitro*. CXCL12 is present in ascites and is expressed by peritoneal mesothelial cells, thus explaining that in part, migration and attachment of ovarian cancer cells to the peritoneum are facilitated by CXCL12–CXCR4 interactions. Ablating this interaction with a CXCR4 antagonist, AMD3100, has been shown to inhibit migration in vitro [53].

While there is some literature on CRAds for ovarian cancer therapy [23], [29], [30], [31], [32], [34], [54], [55], [56], there is very limited research on the usage of an armed CRAd. We report here for the first time, evaluation of a CRAd delivering a noncytotoxic gene for the treatment of disseminated ovarian cancer. It is conceivable that with further refinements in terms of vector targeting, therapeutic dose, multiple dosing schedule and usage with adjuvant chemotherapy or radiotherapy, therapeutic efficacy can be significantly enhanced while minimizing deleterious effect on the immune system.
